# Supplementary material for: Standardized protocol for laboratory rearing and breeding of the Lymnaeidae snail, Radix natalensis (Krauss, 1848)
Source: PLoS One. 2025 Oct 27;20(10):e0335640. doi: 10.1371/journal.pone.0335640 (PMC12558528; doi:10.1371/journal.pone.0335640)
Supplement: Attachment — Submitted filename: Rebuttal letter Addressing Reviewers commemts.docx [file pone.0335640.s004.docx]

Agrippa Dube. Msc. PhD Student

School of Nursing and Public Health

College of Health Sciences

Howard College Campus

University of KwaZulu-Natal

Durban 4001, South Africa

adube168@gmail.com

The Editor-in-Chief

PLoS One

Dear Sir/Madam,

**Ref**: Standardized protocol for laboratory rearing and breeding *Radix natalensis*: Krauss, 1848 (Manuscript Number: PONE-D-25-05169).

We acknowledge receipt of the reviews’ comments for the manuscript submitted to your journal for publication. Please find below a point-by-point rebuttal of the comments that were addressed.

Best,

Agrippa Dube.

| Line No | | | Reviewers' comments | Author's response | | Line edited/ where? | |
| --- | --- | --- | --- | --- | --- | --- | --- |
| **Reviewer 1** | | | | | | | |
| **Methodology** | | | | | | | |
| 1 | | | As I can understand the design of the experiment, the group A was reared under conditions most similar to the natural one for R. natalensis. In this case, this group must be designated as the ‘control’ one, and all discussions of the results must be given as a comparison between the control groups and two other; | We have conceded to the point and hence the experimental control is now Group A which simulates the external environment where the parent snails were collected from. | | Line 134, and Table 1, line 168 | |
| **Discussion** | | | | | | | |
| 2 | | | The reasons why the control group shows the worst performance (mortality, survival, fecundity, etc.) are not clear. The authors give reference to a number of papers, but these explanations are hardly applicable to laboratory conditions. For example, the authors cite several probable causes of low fecundity and survival, based on literary data: environmental changes, the presence of parasites, and inter and intraspecific competition between the snails and other aquatic microfauna. However, neither interspecific competition nor environmental changes are observed in a laboratory-reared culture consisting of individuals of the same species. I suppose that these causes should be deleted, and, instead, the authors have to try to explain, why in their experiment the control group was the worse. | We have reconsidered our argument for poor performance of the control group and revised our discussion accordingly. The natural water used for breeding could have had parasites that we did not screen for that may have interfered with the monitored parameters [1]. The natural feed was inferior, particularly for protein content, to artficial feed used for other groups. Under natural conditions there is continuous repleshnment of nutrients which was not the case for our experiment. In fact the quality of water could have deteriorated during the period it was kept in the laboratory. So overall, the conditions that Group A snails were subjected to were not the best for a laboratory set up and hence the observed poor performance. | | Lines 227-239 | |
| 3 | | | 3. An important recent paper on the laboratory rearing of lymnaeid snails has been overlooked by the authors: Dreyfuss, G., Vignoles, P., Rondelaud, D., Sánchez, J., Vázquez, A.A. (2023). Laboratory Cultures of Lymnaeidae for Parasitological Experiments. In: Vinarski, M.V., Vázquez, A.A. (eds) The Lymnaeidae. Zoological Monographs, vol 7. Springer, Cham. <https://doi.org/10.1007/978-3-031-30292-3_14> | We appreciate the suggestion to include an important publication we had missed. Thus, the article by Dreyfuss, G., Vignoles, P., Rondelaud, D., Sánchez, J., Vázquez, A.A. (2023). Laboratory Cultures of Lymnaeidae for Parasitological Experiments. In: Vinarski, M.V., Vázquez, A.A. (eds) The Lymnaeidae. Zoological Monographs, vol 7. Springer has been cited in the revised manuscript: (Lymnaeid snails are major transmitters of trematodes worldwide [2]). | | lines 67, 94 and 217 | |
|  | | |  |  | |  | |
| **Independent Review Report, Reviewer 2** | | | | | | | |
| **Title** | | | | | | | |
|  | | | I would delete “Research”. I don’t understand the meaning. | We have removed the word “Research” from the title in the revised manuscript | | Title page 1(lines 1-2) | |
|  | | | Add the name of the family of this freshwater snail species. | The family name” Lymnaeidae” has been added to the title to read, “Standardized Protocol for Laboratory Rearing and Breeding of the lymnaeidae snail, *Radix natalensis* Krauss, 1848) | | Title Page 1(1-2) | |
| **Abstract** | | | | | | | |
|  | | | The abstract needs to be restructured into a single paragraph without line breaks. Its current organization is unclear, and it should focus solely on presenting the main results concisely. Given the length of the manuscript, the abstract is too long and should be shortened. | We acknowledge the queries raised. We have shortened the abstract and restructured it, focusing on the main results in a concise way. | | Page 2 (Lines 32-57) | |
| **Background** | | | | | | | |
|  | | | 1. First paragraph – Provide general context on the rearing of lymnaeid snails and why laboratory breeding is essential for epidemiological studies. | A justification for rearing snails for laboratory experiments has been provided, highlighting the importance of such experiments in epidemiological studies | | (Lines 64-91) | |
|  |  | | | | | |  |
|  | | | 2. Second paragraph – Highlight the lack of research on Radix natalensis  and, | We acknowledge this comment. The lack of research on *Radix natalensis* was highlighted in the revised manuscript (Little work has been done on the breeding of *R. natalensis* snails for further laboratory experimental work. To the best of our knowledge, three experimental works have been previously done to determine the influence of *F. gigantica* on the life history traits of *R. natalensis* in East and West Africa [3-5]) | | Lines 90-93 | |
|  |  |  | Why studies on this species are particularly necessary  . | We addressed the issue of the importance of the research on *R. natalensis* (*R. natalensis* is a major Intermediate host snail (IHS) of fascioliasis, a disease of public and veterinary health importance that affects livestock and humans in the tropics and subtropics regions worldwide [6-9]) | | Lines 82-89 | |
|  |  |  | Discuss the impact of the disease it transmits, including its burden on livestock and human populations. | The impact of human and animal fascioliasis was discussed in the revised manuscript. (In livestock, infection leads to animal weight loss, reduced productivity, and fertility, and reduced milk production leading to economic losses [10]. | | Lines 100-102 | |
|  |  |  | Are there reported cases of fascioliasis in cattle or humans? If so, they should be mentioned to emphasize the relevance of studying this species | Cases of cattle and human fascioliasis were mentioned in the revised manuscript. (Bovine fascioliasis is of concern in livestock production in South Africa [11, 12]. In South Africa, human fascioliasis may not be a burden, but 2 cases were reported in Gauteng (1956) and Western Cape Province (1964) [13]) | | Lines 102-103 | |
|  | | | 3. Third paragraph – Clearly state what the authors did in this study, their objectives, and the methodology they used | We addressed the issues raised in the revised manuscript. ( To enable current and future laboratory mass breeding of *R. natalensis* for laboratory experiments, we report results from a cost-effective standardized rearing and breeding protocol for mass-producing F_1_ *R. natalensis* snails). | | 106-108 | |
|  |  |  | The introduction should be rewritten to improve readability and clarity, making it easier for the reader to follow the authors' reasoning. | Introduction has been revised to improve readability and clarity. | | Lines 60-87 | |
|  |  |  | Additionally, at one point, the authors refer to a study where experimental work with this species was conducted in the laboratory,However, it is unclear who conducted that study and how it relates to the present manuscript. If it is relevant, more context should be provided to clarify its significance. | The previous laboratory work referred to in previous version has been presented indicating the authors, in Africa were carried by Bitakaramire ( 1969) in Kenya [5], Henry Madsen (1981) in Kenya [14] and Adediran (2013) in Nigeria [4], and non on the *Radix natalensis* strain found in Subtropical regions of South Africa, (To the best of our knowledge, three experimental works have been previously done to determine the influence of *F. gigantica* on the life history traits of *R. natalensis* in East and West Africa [3-5]). | | Lines 90-94 | |
|  |  |  |  |  | |  | |
|  |  |  | The introduction should provide broader context on lymnaeid snails rather than focusing too much on Radix natalensis alone. While there is extensive research on other lymnaeid species, this particular species has not been studied as much, despite its significant epidemiological importance in this region of the world. | In the introduction we have also placed emphasis on lymnaeid snails rather *R. natalensis* alone. (Globally, about 20 species of lymnaeid snails have been described as potential IHS of *Fasciola* sp. [15, 16]. *Galba truncatula*, *Austropeplea (Lymnaea) tomentosa,* *Lymnaea viatrix, Lymnaea viridis, Galba cubensis and* *Pseudosuccinea columella* are some of the common IHS of *F. hepatica* in the temperate regions of Europe, Asia, Australia and Americas [15] while *Radix auricularia, Radix acuminata, Pseudosuccinea columella* and *Radix natalensis* transmit *F. gigantica* in the tropical and subtropical religions of Asia and Africa [15, 17]) | | Line 63-67 | |
| **Material and Methods** | | | | | | | |
|  | | | | | | | |
|  | | | The most significant issue with this study is the lack of replication. Replicates are crucial in scientific experiments because they allow for the assessment of variability within treatments and ensure the reliability and generalizability of the results. In this study, the authors used 30 individuals per aquarium and assigned each aquarium a different treatment, with only 3 aquariums in total (and thus 3 treatments). This approach does not account for natural variation within each treatment group. Without replication, the results are less robust, and any observed effects may be due to random chance or uncontrolled environmental factors. Replicating treatments would provide more reliable data and allow for meaningful statistical comparisons, ultimately strengthening the conclusions of the study. | We acknowledge this comment: We have clarified the issue of replicates by stating that ninety snails were selected and assigned to three treatment Groups: A, B, and C. Each experimental group had 10 containers, each containing 3 snails thus making it 30 snails per experimental group.Thus, in our experiment, the containers were the replicates. This would translate to each treatment having 9 replicates. Group A is the control experiment, which simulates the external environment where snails were collected. | | Lines 125-135 | |
| **Results** | | | | | | | |
|  | | | Delete decimals in percentages. Use one decimal for values under 10% or over 90%. See | The authors addressed the issue raised by deleting the decimal places as advised. Percentage mortality \| 67 \| 13 \| 20 | | Table 1. line 163 | |
|  | | | Figure 1 shows a deviation that I do not understand how it was calculated. | The issue of deviation in Fig _1_ was addressed in the revised manuscript:   | | Fig 1, line 170 | |
| **DISCUSSION** | | | | | | | |
|  | | | Given that there are no replicates for the treatments, I find it difficult to believe that the differences observed in the statistical estimates have biological relevance. Replicates are essential to ensure the reliability and generalizability of the results. Without them, it is challenging to draw meaningful conclusions about the true effects of the treatments. | We acknowledge this comment and our limitation clearly explaining the number of replicates in our study. We have now clarified that each experimental group had 30 snails and these 30 snails were put in 10 containers. We have also indicated that each container had 3 snails. Thus, each treatment group had 9 replicates and one main container, making it 10 containers per experimental group. | | Line 125-135 | |
|  | | To improve the experiment, the authors should include multiple replicates per treatment. For example, they could use at least three aquariums per treatment, each with 30 snails, to account for variability and ensure more reliable results. This way, the effects of the treatments can be more accurately assessed and statistically analyzed, leading to stronger conclusions. | | | We acknowledge this important observation by the reviewer. One of the major challenges we faced was in finding adequate snail numbers to allow us to carry out a large breeding experimental study. A study by Hadebe [18], in review study by[10] and research article [19] acknowledge that *R. natalensis* is becoming less abundant in South Africa [20]. In our study, we managed to collect 120 wild snails from only 2 out of 45 sites vsited. However, in the process of acclimatization to labaoratory conditions before the start of the experiment, we lost 30 snails leaving us with 90 snails. These were the available snails for the experiment that has been reported in this paper. | Line 123 | |
|  | |  | | |  |  | |

**References**

1.	Van Someren VD. The habitats and tolerance ranges of Lymnaea (Radix) caillaudi, the intermediate snail host of liver fluke in East Africa. The Journal of Animal Ecology. 1946:170-97.

2.	Dreyfuss G, Vignoles P, Rondelaud D, Sánchez J, Vázquez AA. Laboratory cultures of Lymnaeidae for parasitological experiments. The Lymnaeidae: A Handbook on Their Natural History and Parasitological Significance: Springer; 2023. p. 395-414.

3.	Madsen H, Stauffer JR. Zoonotic trematode infections; their biology, intermediate hosts and control. 2022.

4.	Adediran O, Uwalaka E. Laboratory breeding of Lymnaea natalensis (Krauss, 1848), intermediate host of Fasciola gigantica (Cobbold, 1856). 2013.

5.	Bitakaramire P. Lymnaea natalensis laboratory culture and production of Fasciola gigantica metacercariae. Parasitology. 1968;58(3):653-6.

6.	Jaja IF, Mushonga B, Green E, Muchenje V. Seasonal prevalence, body condition score and risk factors of bovine fasciolosis in South Africa. Veterinary and Animal Science. 2017;4:1-7.

7.	Mpisana Z, Jaja IF, Byaruhanga C, Marufu MC. Body condition scores, fluke intensity, liver pathology, and carcass quality of different dairy cattle genotypes infected with Fasciola species at high throughput abattoirs in South Africa. Parasitology Research. 2022;121(6):1671-82. doi: 10.1007/s00436-022-07504-9.

8.	DP A, MB C, CC B, DM D, OS PJ, BR A, et al. Distribution and factors associated with Fasciola hepatica infection in cattle in the south of Espírito Santo State, Brazil. Journal of Venomous Animals and Toxins including Tropical Diseases. 2011;17:271-6.

9.	Rashid M, Rashid MI, Akbar H, Ahmad L, Hassan MA, Ashraf K, et al. A systematic review on modelling approaches for economic losses studies caused by parasites and their associated diseases in cattle. Parasitology. 2019;146(2):129-41.

10.	Ngcamphalala P, Malatji M, Mukaratirwa S. Geography and ecology of invasive Pseudosuccinea columella (Gastropoda: Lymnaeidae) and implications in the transmission of Fasciola species (Digenea: Fasciolidae)–a review. Journal of helminthology. 2022;96:e1.

11.	Nukeri S, Malatji MP, Sithole MI, Ngcamphalala PI, Nyagura I, Tembe D, et al. Infection rates of Fasciola spp. in cattle slaughtered at 13 abattoirs in six of nine provinces of South Africa. Food and Waterborne Parasitology. 2025:e00260.

12.	Olaogun SC. Knowledge, practices, and epidemiology of bovine fasciolosis in smallholder farming areas of South Africa: University of Pretoria (South Africa); 2022.

13.	Black J, Ntusi N, Stead P, Mayosi B, Mendelson M. Human fascioliasis in South Africa. South African Medical Journal. 2013;103(9):658-9.

14.	Madsen H, Monrad J. A method for laboratory maintenance of Lymnaea natalensis and for mass production of Fasciola gigantica metacercariae. The Journal of Parasitology. 1981;67(5):735-7.

15.	Dube A, Kalinda C, Manyangadze T, Mindu T, Chimbari MJ. Effects of temperature on the life history traits of intermediate host snails of fascioliasis: A systematic review. PLOS Neglected Tropical Diseases. 2023;17(12):e0011812. doi: 10.1371/journal.pntd.0011812.

16.	Claxton J, Sutherst J, Ortiz P, Clarkson M. The Effect of Cyclic Temperatures on the Growth ofFasciola hepaticaandLymnaea viatrix. The Veterinary Journal. 1999;157(2):166-71.

17.	Mas-Coma S, Valero MA, Bargues MD. Human and animal Fascioliasis: origins and worldwide evolving scenario. Clinical Microbiology Reviews. 2022;35(4):e00088-19.

18.	Hadebe MI, Manyangadze T, Kalinda C, Chimbari MJ. Factors contributing to the abundance and spatial distribution of the invasive intermediate host snail (Pseudosuccinea columella) in uMgungundlovu district, KwaZulu-Natal, South Africa. BMC Veterinary Research. 2025;21(1):254. doi: 10.1186/s12917-025-04703-1.

19.	De Kock K, Joubert P. Suitability of tropical fish foods for laboratory culture of four species of freshwater snails acting as intermediate hosts for economically important helminth parasites in South Africa. Southern African Journal of Aquatic Science. 1989;15(1):91-102.

20.	Malatji MP, Lamb J, Mukaratirwa S. Molecular characterization of liver fluke intermediate host lymnaeids (Gastropoda: Pulmonata) snails from selected regions of Okavango Delta of Botswana, KwaZulu-Natal and Mpumalanga provinces of South Africa. Veterinary Parasitology: Regional Studies and Reports. 2019;17:100318.
