# Supplementary material for: Standardized protocol for laboratory rearing and breeding of the Lymnaeidae snail, Radix natalensis (Krauss, 1848)
Source: PLoS One. 2025 Oct 27;20(10):e0335640. doi: 10.1371/journal.pone.0335640 (PMC12558528; doi:10.1371/journal.pone.0335640)
Supplement: Attachment — Submitted filename: Response to Reviewers.docx [file pone.0335640.s005.docx]

Agrippa Dube. Msc. PhD Student

School of Nursing and Public Health

College of Health Sciences

Howard College Campus

University of KwaZulu-Natal

Durban 4001, South Africa

adube168@gmail.com

The Editor-in-Chief

PLoS One

Dear Sir/Madam,

**Ref**: Standardized Protocol for Laboratory Rearing and Breeding of the Lymnaeidae snail, Radix natalensis (Krauss, 1848). (Manuscript Number: PONE-D-25-05169).

We acknowledge receipt of the reviewers’ comments for the manuscript submitted to your journal for publication. Please find below a point-by-point rebuttal of the comments that were addressed. We noted that some of the comments made do not apply to our manuscript, as the indicated text and figures do not correspond with what is in our original manuscript. We have highlighted in yellow where such comments were made.

Best,

Agrippa Dube.

| Reviewers' comments | | Authors’ response | Line edited/ where? |
| --- | --- | --- | --- |
| **Editorial comments** | |  |  |
| Please amend the title either on the online submission form or in your so that they are identical. | | We thank you for bringing this to our attention. We acknowledge the query and confirm that the title has been amended in the online submission form to correspond with the revised manuscript. | in the online submission form |
| Your abstract cannot contain citations. Please remove any existing citations from the abstract section. You may only include citations in the body text of the manuscript, and please ensure that they remain in ascending numerical order on first mention | | We acknowledge the query raised. The citation was removed from the abstract and placed in the background section of the revised manuscript. | Lines 86-88 |
| Your ethics statement should only appear in the Methods section of your manuscript. If your ethics statement is written in any section besides the Methods, please delete it from any other section. | | Thank you. The ethics statement was placed in the methodology section of the revised manuscript. | Lines 143-147 |
|  | |  |  |
| **Reviewer 2** | | | |
| **Absract** | | | |
| • “Group A, containers were filled” → should be “Group A containers were filled” | | We acknowledge the query. In the revised manuscript, the statement has been corrected to read: ‘Group A containers were filled …’ | Line 41 |
| “0.85±0.22egg masses” → insert spaces: “0.85 ± 0.22 egg masses” | | Thank you. In the revised thesis, spaces have been inserted between figures as suggested. | Lines 47, 48 & 51 |
| • “mass breading” → correct to “mass breeding” | | We acknowledged the query raised. The term “ mass breading” was corrected to “mass breeding” in the revised manuscript. | Line 53 |
| • Sentence structure is dense; breaking long sentences would improve readability. | | We acknowledged the query raised. We have addressed the raised issue throughout the revised manuscript. | Revised manuscript |
| Ensure consistency in terminology: use either “F1 offspring” or “F1 progenies” throughout the manuscript. | | Thank you. For consistency, the authors have adopted the term ‘offspring’ throughout the revised manuscript. | Line 43 |
| **Introduction** | | | |
| • “Altitudinal differences suggest that understanding and evaluating breeding feasibilities…” → Does this refers to differences in altitude between previous studies and the current one? | | We acknowledged the good observation. We addressed the query by restructuring the statement to “Earlier studies provide important insights on how to breed snails using dried lettuce, trout pellets, and crushed chicken eggshells as snail feed, as well as the use of dechlorinated water in researches conducted in Kenya and Nigeria by [1, 2]. However, differences in altitude between the regions where these studies were conducted and our study area suggest that assessing the breeding feasibility of locally available snails would improve preparedness for fascioliasis outbreaks, particularly in Southern Africa.” | Lines 86-88 |
| • “Led to having inaccurate and not reproducible results” → replace by “led to inaccurate and non-reproducible results” | | We acknowledge the query. In the revised manuscript, the sentence has been reconstructed to read: “An earlier study on the breeding of *Bulinus globosus* suggested that the use of field-collected snails in laboratory studies may lead to inaccurate and non-reproducible results due to changes in conditions and inadequate acclimatization time.” | Lines 99-102 |
| • “Religions” → should be “regions” | | The typographical error has been corrected by replacing the word ‘religions’ with ‘regions’ in the revised manuscript. | Line 68 |
| • “Acclimazation” → replace by “acclimatization” | | Thank you. We have corrected the spelling error by replacing the word “Acclimazation” with “acclimatization” in the revised manuscript. | Line 102 |
| • “Conducting laboratory mechanistic experiments is in raising a snail colony…” → rephrase for clarity: “A key requirement for conducting mechanistic experiments is raising a clean, laboratory-bred snail colony…” | | We acknowledge the query. In the revised manuscript, the sentence has been restructured to read: “A key requirement for conducting mechanistic experiments is raising a clean, laboratory-bred snail colony that is free from field trematode infection.” | Lines 73-75 |
| • The following sentence should be revised: “Breeding of snails is also important in drug vaccine research [10]. Snails are used in trials to evaluate new anthelmintics medications and biological control approaches, such as vaccines targeting different parasite life cycle stages [11].” Vaccines against fascioliasis are currently being developed for livestock (sheep and cattle), not for snails. Snails are not the target of vaccination, and immune-based interventions in mollusks are not widely studied or applied. | | We acknowledge the question raised. We have combined the sentences to make one that reads: “Breeding of snails is also important because snails are used as hosts for research into drugs and vaccines, such as those for parasitic diseases like trematodiasis [3, 4].” | Lines 82 and 83 |
|  |  |  |  |
| **Materials and Methods** | | | |
|  | | | |
| • Replace “R. natalensis snails for the study were identified…” by “Snails were identified using morphological keys from Brown (1994) and Appleton (1996)...” | | We acknowledged the suggestion. The revised sentence now reads: “Snails for the study were identified using morphological identification keys designed by Brown (1994) [5] and Appleton (1996) [6].” | Lines 117 & 118 |
| • “During the process of acclimatization to laboratory conditions and before being used…” replace by “During acclimatization to laboratory conditions, 30 snails died before allocation to treatment groups.” | | Thank you. In the revised manuscript, the statement reads: “However, during acclimatization to laboratory conditions, 30 snails died before being allocated to treatment groups.” | Lines 124 &125 |
| • “10 transparent containers with 3 snails each…” replace by “Each treatment group consisted of 10 transparent 2L plastic containers (26.65 cm × 19.3 cm × 6.45 cm), with three snails per container, totaling 30 snails per group.” | | We acknowledged the query raised, and we have rephrased the statement in the revised manuscript to read: ‘Each treatment group consisted of 10 transparent 2L plastic containers (26.65 cm × 19.3 cm × 6.45 cm), with three snails per container, totaling 30 snails per group.” | Lines 128-130 |
| • Treatments description: Improve clarity and reduce repetition: Group A (control): fed Elodea weed powder; Cyperus papyrus twigs used as oviposition substrate; field-collected water used; Group B: fed dry lettuce, fish flakes, and crushed eggshells; spring water used; and Group C: fed algae wafers and trout pellets; dechlorinated tap water used (left to stand for four days). All groups were fed three times per week; water was changed at feeding to prevent waste buildup.” | | We acknowledged the query raised. In the revised manuscript we have revised the description to: “Group A (control): fed with *Elodea* weed powder; *Cyperus papyrus* twigs provided oviposition substrate; field-collected water was used; Group B: fed with dry lettuce, fish flakes, and crushed eggshells; spring water was used; and Group C: fed with algae wafers and trout pellets; dechlorinated tap water was used (left to stand for four days). All groups were fed three times per week and water was changed at feeding to prevent waste buildup.” | Lines 130-134 |
| • “Egg masses… were removed and transferred to new containers.” Replace by “Egg masses laid on substrates or container walls were carefully removed and transferred to new containers containing the same water type as the original group to ensure  consistency.” | | Thank you for the good suggestion. We addressed the query by reconstructing the sentence to read: “Egg masses laid on substrates or container walls were carefully removed and transferred to new containers containing the same water type as the original group to ensure consistency”. | Lines 135-137 |
| • “Ambient and water temperatures were measured daily…” replace by “Ambient and water temperatures were monitored daily and maintained at 26 ± 1 °C (air) and 22 ± 1 °C (water), respectively.” | | Thank you. The authors adopted the suggested sentence in the revised manuscript. | Lines 137 &138 |
|  | |  |  |
| **Results** | | | |
| • “Snail egg-laying behaviour and hatching success were affected…” →replace by something like “Egg-laying behavior and hatching success were influenced by food type, water quality, and oviposition substrate.” | | We acknowledge the query raised. We thoroughly reviewed the manuscript but did not find the statement indicated for correction, as it was not part of our results. |  |
| • “The snails in group A… laid the highest number of egg masses…” replace by “Group A snails (fed Elodea weed powder, with papyrus twigs and field water) produced the most egg masses (n = 91), followed by Group B (n = 57) and Group C (n = 24).” | | We acknowledge the query. The authors have addressed the language aspect only, as the results suggested or the figures represented in the comments do not correspond with those observed in the current study or reported in the original manuscript. In fact, in our manuscript group A had the least number of eggs. | Lines 161-163 |
| • “Overall, 197 egg masses comprising 1076 eggs…” replace by “A total of 197 egg masses containing 1,076 eggs were laid across all groups: Group A accounted for 46.2% of eggs, Group B for 33.3%, and Group C for 20.3%.” | | We acknowledged the query. The authors have addressed the language aspect only, as the results suggested or the figures represented in the comments do not correspond with those observed in the current study or reported in the original manuscript. | Lines 166-168 |
| • “Hatching rates were highest in group A…” replace by “Hatching success was highest in Group A (88.7%), moderate in Group B (75.6%), and markedly lower in Group C (41.9%).” | | We acknowledged the query. The authors have addressed the language aspect only, as the results suggested or the figures represented in the comments do not correspond with those observed in the current study or reported in the original manuscript**.** |  |
| **Discussion** | | | |
| • “This study demonstrates the importance…” replace by something like “Our findings highlight the critical role of laboratory conditions—particularly food type, water quality, and oviposition substrate—in maintaining Radix natalensis and optimizing its reproductive output.” | | We acknowledge the query. The authors revised the statement to read: ‘Our findings highlight the critical role of laboratory conditions, particularly food type, water quality, and oviposition substrate in maintaining wild-caught F_0_ generation *R. natalensis* and optimizing its reproductive output F_1_ generation’ in the revised manuscript. | Lines 197-199 |
| • “Snails fed Elodea…” replace by “Elodea-fed snails in field water exhibited significantly higher reproductive output, suggesting this combination most closely mimics their natural environment.” | | We acknowledge the query and have addressed the language aspect accordingly. However, the areas suggested for revision in the comments do not correspond with those presented in the original manuscript. | Lines 219 - 221 |
| • “The low egg production and hatching rate in group C…” replace by “The reduced reproductive success observed in Group C likely reflects suboptimal nutritional and water conditions, as neither the artificial feed nor the dechlorinated tap water closely replicate the snails’ natural habitat.” | | We acknowledge the query raised. However, the suggested revisions were not addressed, as they do not align with the findings of the current research. |  |
| • “This shows the need for care when designing…”. I suggest “These results underscore the importance of carefully selecting husbandry conditions in experimental protocols involving R. natalensis, particularly for studies on parasite transmission or life cycle maintenance.” | | We acknowledge the query raised. The author has incorporated the suggested statement as a concluding line. | Lines 235 and 237 |

**References**

1. Madsen H, Monrad J. A method for laboratory maintenance of *Lymnaea natalensis* and for mass production of *Fasciola gigantica* metacercariae. The Journal of Parasitology. 1981;67(5):735–7.

2. Adediran O, Uwalaka E. Laboratory breeding of *Lymnaea natalensis* (Krauss, 1848), intermediate host of *Fasciola gigantica* (Cobbold, 1856). 2013.

3. Dhiman V, Pant D. Human health and snails. Journal of Immunoassay and Immunochemistry. 2021;42(3):211–35.

4. Svigruha R, Fodor I, Padisak J, Pirger Z. Progestogen-induced alterations and their ecological relevance in different embryonic and adult behaviours of an invertebrate model species, the great pond snail (*Lymnaea stagnalis*). Environmental Science and Pollution Research. 2021;28:59391–402.

5. Brown DS. Freshwater snails of Africa and their medical importance: CRC press; 1994.

6. Appleton CC. Freshwater Molluscs of Southern Africa: With a Chapter on Bilharzia and Its Snail Hosts: University of KwaZulu Natal Press; 1996.
